# Elemental classification of the tusks of dugong (Dugong dugong) by HH-XRF analysis and comparison with other species

**DOI:** 10.1038/srep46167

**Published:** 2017-04-07

**Authors:** Korakot Nganvongpanit, Kittisak Buddhachat, Promporn Piboon, Thippaporn Euppayo, Patcharaporn Kaewmong, Phaothep Cherdsukjai, Kongkiat Kittiwatanawong, Chatchote Thitaram

**Affiliations:** 1 Animal Bone and Joint Research Laboratory, Department of Veterinary Biosciences and Public Health, Faculty of Veterinary Medicine, Chiang Mai University, Chiang Mai 50100, Thailand; 2 Department of Biology, Faculty of Science, Naresuan University, Phitsanulok 65000, Thailand; 3 Phuket Marine Biological Center, Phuket 83000, Thailand; 4 Center of Excellence in Elephant Research and Education, Faculty of Veterinary Medicine, Chiang Mai University, Chiang Mai 50100, Thailand

## Abstract

The elemental composition was investigated and applied for identifying the sex and habitat of dugongs, in addition to distinguishing dugong tusks and teeth from other animal wildlife materials such as Asian elephant (*Elephas maximus*) tusks and tiger (*Panthera tigris tigris*) canine teeth. A total of 43 dugong tusks, 60 dugong teeth, 40 dolphin teeth, 1 whale tooth, 40 Asian elephant tusks and 20 tiger canine teeth were included in the study. Elemental analyses were conducted using a handheld X-ray fluorescence analyzer (HH-XRF). There was no significant difference in the elemental composition of male and female dugong tusks, whereas the overall accuracy for identifying habitat (the Andaman Sea and the Gulf of Thailand) was high (88.1%). Dolphin teeth were able to be correctly predicted 100% of the time. Furthermore, we demonstrated a discrepancy in elemental composition among dugong tusks, Asian elephant tusks and tiger canine teeth, and provided a high correct prediction rate among these species of 98.2%. Here, we demonstrate the feasible use of HH-XRF for preliminary species classification and habitat determination prior to using more advanced techniques such as molecular biology.

The dugong (*Dugong dugon*, Müller), or sea cow, is one of four herbivorous marine mammal species surviving in the family Dugongidae, order Sirenia. The dugong grazes exclusively on seagrass and is thus limited to coastal habitats where there is an abundance of seagrass meadows^1^. Currently, dugong populations are persistently declining due to anthropogenic causes, including illegal hunting, habitat degradation and fishing-related fatalities^2^. The dugong is a vulnerable species and has been placed in Appendix I of the Convention on International Trade in Endangered Species (CITES).

Unlike other mammalian species, the study of dugong teeth is not well-established. The dugong possesses a pair of tusks, formed by the first upper incisor teeth in both males and females, that are used as cutting instruments for foraging^3^. Sexual dimorphism is exhibited in dugongs by tusk eruption in males after puberty due to increased testosterone levels^4^,^5^,^6^, and also rarely in females older than 40 years^5^. Dugong tusks have been used for age estimation based on dentinal growth layer groups (GLGs) and cheek-tooth development using a multiple logarithmic regression model^4^,^7^,^8^,^9^, as well as puberty prediction based on erupted teeth^4^,^5^. A relationship was also demonstrated between behavioral characteristics and testosterone content in tusks^6^. Furthermore, the results of an osteological study and skull measurements of dugongs from India suggested an osteological similarity between dugongs from India and the Red Sea, whereas a discrepancy in skull measurements was observed in the different regions^10^. From a literature review, we found only two publications reporting on elements in dugong tusks. A study in 1979 was able to identify real dugong tusks using strontium (Sr) content and microstructure^11^, while a second study in 1997 demonstrated the difference in elemental distribution in dentinal growth layer groups (GLGs) in female dugong teeth^12^. However, the latter study had a limited number of samples, i.e. a single female dugong.

In recent years, the elemental content in biological tissues (i.e. bones, teeth, soft tissues or body fluids) of a particular mammal species has been studied for a wide range of purposes, such as tissue structure^13^,^14^, the role of elements in physiology^15^,^16^,^17^, contamination by environmental pollution^18^,^19^, and forensic science^20^,^21^,^22^,^23^,^24^,^25^. Many techniques are used for element detection, such as inductively coupled plasma mass spectrometry (ICP-MS)^26^, atomic absorption spectroscopy (ASS)^27^ and X-ray fluorescence (XRF)^28^,^29^; each technique has both advantages and disadvantages. The selection of a suitable technique depends on the purpose of the investigation.

Little is known about the elemental composition of dugong tusks. A previous study in female dugongs analyzed elements in the 55 annual incremental longitudinal sections of tusks, and reported the presence of nine elements: barium (Ba), lithium (Li), sodium (Na), calcium (Ca), iron (Fe), magnesium (Mg), phosphorus (P), strontium (Sr) and zinc (Zn)^12^. Most elements revealed both long-term trends and shorter, year-to-year, variation. Nevertheless, no significant correlation between dentinal GLGs and elements was observed. A previous study that was conducted using laser ablation–inductively coupled plasma–mass spectrometry to construct two-dimensional maps of trace elements in human teeth reported the heterogeneity of these elements in teeth^30^. In this study, a handheld X-ray fluorescence analyzer (HH-XRF) was employed as a tool for detecting elements in our samples because this technique is non-invasive. All samples used in this study were under the authority of museums or belonged to private owners who did not allow any destruction of the samples. HH-XRF has been shown to be an appropriate technique for detection of elements in animal tissues such as horn/antler^24^, bone^21^,^24^,^31^ or teeth/tusks^22^,^23^,^25^ for either biological or forensic purposes. For example, analysis of elemental data by discriminant analysis and logistic regression models has been successful in identifying the origin of elephant tusks, i.e. whether Asian or African^25^, and authenticating real elephant tusks^23^, leading to a high accuracy rate. In addition, analysis of the elemental composition by stepwise discriminant analysis could correctly discriminate between human and non-human teeth^22^. Although modern techniques have high robustness and reliability – e.g. molecular methods, including polymerase chain reaction (PCR) and sequencing that have been used for species identification^32^,^33^ and sex determination of dugongs and other species^34^,^35^ – they are time-consuming, expensive and require considerable technical expertise and laboratory facilities. Elemental analysis by HH-XRF is not only less time-consuming, but also does not involve destruction of the sample (invasive technique); moreover, no laboratory work is required, making it suitable for primary screening in a field study prior to further assessment by a more advanced technique^22^,^23^,^25^,^29^.

In coastal regions of Thailand, dugong meat is considered as food, and various body parts of the dugong, such as tusks and bones and even tears, are believed to possess medicinal or protective benefits^36^; the tusk is regarded as the most valuable for protective benefits^37^. Nowadays, there are five laws relevant to dugong and seagrass (their main food source) conservation in Thailand: (i) Fisheries Act, B.E. 2490 (1947), (ii) National Park Act, B.E. 2504 (1961), (iii) Export and Import of Goods Act, B.E. 2522 (1979), (iv) Wildlife Preservation and Protection Act, B.E. 2535 (1992) and (v) CITES^37^. All laws prohibit the killing, taking, possessing and trading of dugongs or body parts of dugongs in Thailand, and CITES bans these activities internationally. However, trade in dugong parts such as tusks is difficult to detect. For this reason, the use of elemental analysis needs to be developed as a tool for identifying and differentiating dugong tusks from other valuable animal wildlife materials: Asian elephant (*Elephas maximus*) tusks and tiger (*Panthera tigris tigris*) canine teeth.

## Material and Methods

### Samples

Dugong teeth samples were obtained from the Reference Collection, Phuket Marine Biological Center (PMBC), Phuket, Thailand. The use of animal bones from the Animal Anatomy Museum, Faculty of Veterinary Medicine, Chiang Mai University, did not require approval by the Animal Ethics Committee. All teeth samples were dry, and maintained at room temperature. They were immediately cleaned upon death, but otherwise had not been altered (burned or buried). None of the samples exhibited pathological lesions or disease conditions. A total of 43 individual dugong tusks (male = 17, female = 25, unknown sex = 1) were included in this study. In addition, 60 dugong teeth from 30 individuals (2 teeth per animal), 40 dolphin teeth from 10 individuals (4 teeth per animal) (from PMBC), 1 whale tooth (from PMBC), 40 Asian elephant tusks (1 tusk per animal) (from the collection of a private owner, all legally acquired) and 20 tiger canine teeth from 5 individual animals (4 teeth per animal) (from the collection of a private owner, all legally acquired) were also included in the study to compare and classify elements among these species.

### Handheld X-ray fluorescence

Teeth elemental analyses were conducted using a handheld X-ray fluorescence analyzer (HH-XRF) (DELTA Premium; Olympus, Tokyo, Japan), which uses a silicon drift detector, detecting from magnesium (Mg^12^) through bismuth (Bi^83^) on the periodic table. The collimator size was set at 0.3 mm for the analysis area diameter, using the standard mining plus mode. Calibrations were performed each day before the first use of the HH-XRF for sample analysis. Light elements (LE) were those with an atomic number lower than Mg (H^1^–Na^11^), which could not be differentiated as single elements. For each scan (2 min each), the XRF unit was secured in a stand and the sample was placed directly adjacent to the puncture-resistant window of the machine to limit the distance between the detector and sample. Each element was expressed as a percentage. The XRF method was noninvasive, and samples were not manipulated or destroyed in the process of scanning.

### Study design and statistical analysis

To more fully understand the elemental composition in dugong tusks and teeth, this research was categorized into seven studies. The samples used in each study were different, depending on the recorded history of samples. The results of each element were presented as a percentage. Moreover, the ratio between Ca and P was calculated in all studies because they are the major inorganic components of calcium phosphate [Ca_3_(PO_4_)_2_]; this interacts with calcium hydroxide [Ca(OH)_2_] to form hydroxyapatite [Ca_10_(PO_4_)_6_(OH)_2_], which can indicate teeth strength^16^,^17^,^38^,^39^.

### Study 1: Elemental analysis comparing the crown and root of dugong tusks

**Figure 1:**
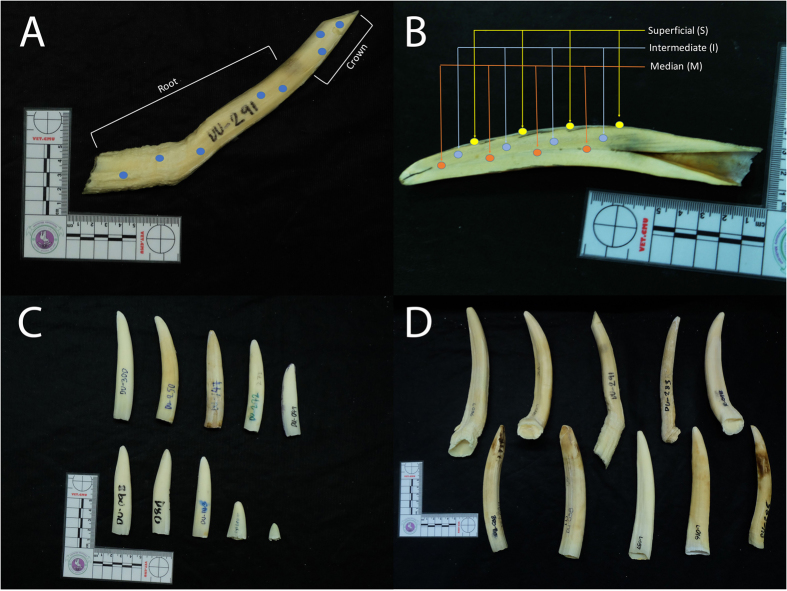
Scanned locations on dugong tusks. (**A**) Schematic of tusk crown and root in study 1. (**B**) Three different tusk layers in study 2. (**C**) Small tusks and (**D**) large tusks in study 3.

This study compared the elemental composition between the crown and root of dugong tusks. In this study, 10 tusks were used as subjects. We scanned the crown (2 or 3 different locations) and root (3 or 4 different locations) of each tusk (Fig. 1A). Elements were presented as a percentage and were compared between the two groups using Mann–Whitney *U* test for nonparametric data analysis.

### Study 2: Elemental analysis comparing different tusk layers

Dugong tusks are large, elongated upper incisor teeth. To study the difference of elemental accumulation between different tusk layers, longitudinal sections of 10 permanent tusks were used as subjects. We scanned the superficial, intermediate and medial layers of each tusk (Fig. 1B). Elements were presented as a percentage and were compared between the three groups by one-way ANOVA, followed by Mann–Whitney *U* test.

### Study 3: Elemental analysis comparing small and large permanent tusks

In this study a total of 39 dugong tusk samples, 15 small tusks (length < 7 cm) and 24 large tusks (length > 7 cm), were used as subjects (Fig. 1C,D). To study the difference of elemental accumulation by tusk size, the surface of each tusk was randomly scanned in 3–5 different locations. Elements were presented as a percentage and were compared between the two groups using Mann–Whitney *U* test for nonparametric data analysis.

### Study 4: Elemental analysis comparing dugong sexes

As is well-established, there is a discrepancy in the metabolism of males and females^17^,^40^,^41^. To study the accumulation of elements between sexes, a total of 42 tusk samples were examined, consisting of 17 tusks from males and 25 tusks from females. The surface of each tusk was randomly scanned in at least 3–5 different locations. Elements were presented as a percentage and were compared between the two groups using Mann–Whitney *U* test for nonparametric data analysis. To establish an equation for sex determination, we compared the elemental composition of male and female dugong tusks through a stepwise discriminant analysis with leave-one-out classification.

### Study 5: Elemental analysis comparing dugong tusks from the Andaman Sea and the Gulf of Thailand

Food and environment can affect the elemental accumulation in the whole body, including mineralized tissues such as bones and teeth^17^,^40^,^41^,^42^. To determine if different habitats cause different elemental composition in dugong tusks, we compared dugong tusks obtained from animals from the Andaman Sea and the Gulf of Thailand. A total of 39 tusk samples were examined, consisting of 31 tusks taken from the Andaman Sea and 7 tusks taken from the Gulf of Thailand. The surface of each tusk was randomly scanned in at least 3–5 different locations. Elements were presented as a percentage and were compared between the two groups using Mann–Whitney *U* test for nonparametric data analysis. To establish an equation for habitat estimation, we compared the elemental composition of dugong tusks from the Andaman Sea and the Gulf of Thailand through a stepwise discriminant analysis with leave-one-out classification.

### Study 6: Elemental analysis comparing dugong tusks, dugong, dolphin and whale teeth

The aim of this study was to analyze the difference in elemental composition of dugong tusks and teeth compared with the teeth of two other marine mammals, dolphin and whale. A total of 43 dugong tusks (n of animals = 43), 60 dugong teeth (n = 30), 40 dolphin teeth (n = 10) and 1 whale tooth (n = 1) were subjected to HH-XRF. The surface of each dugong tusk was randomly scanned in 3–5 different locations, dugong and dolphin teeth in two different locations, and the whale tooth in six different locations. Elements were presented as a percentage and were compared between the four groups using one-way ANOVA, followed by Mann–Whitney *U* test. To establish an equation for species differentiation, we compared the elemental composition of tusks and teeth among dugong, dolphin and whale through a stepwise discriminant analysis with leave-one-out classification.

### Study 7: Elemental analysis comparing dugong tusks, Asian elephant tusks and tiger canine teeth

Elephant tusks were selected as representative of land mammals for purposes of comparison with marine mammals. In addition, tiger (*Panthera tigris tigris*) canine teeth were also included in the study due to their high value in the illegal market, similar to dugong and elephant tusks. A total of 43 dugong tusks (n of animals = 43), 40 male Asian elephant tusks (n = 40) and 20 tiger canine teeth (n = 5) were subjected to HH-XRF. The surface of each sample was randomly scanned in at least 3–5 different locations. To establish an equation for tusk species determination, we compared the elemental composition of dugong tusks, Asian elephant tusks and tiger canine teeth through a stepwise discriminant analysis with leave-one-out classification.

## Results

### Study 1: Elemental analysis comparing the crown and root of dugong tusks

A total of 20 elements plus LE were detected in the crown and root (Table 1). There were seven elements that were significantly higher in the crown compared with the root: titanium (Ti), vanadium (V), manganese (Mn), Fe, silver (Ag), tin (Sn) and antimony (Sb). The Ca/P ratio was higher in the root than in the crown (p > 0.05).

### Study 2: Elemental analysis comparing different tusk layers

A total of 22 elements and LE were detected in three different layers of the tusks (Table 2). Only 18 elements were found in all three layers, including aluminum (Al), silicon (Si), P, sulfur (S), Ca, chromium (Cr), nickel (Ni), copper (Cu), Zn, zirconium (Zr), cadmium (Cd), Ti, V, Mn, Fe, Ag, Sn and Sb. Four elements were found only in some layers: chlorine (Cl) and potassium (K) were not detected in the superficial layer, while tungsten (W) and lead (Pb) were not detected in the intermediate and medial layers of tusks. Three elements (Ni, Cu and Zr) showed a similar content (p > 0.05) in all three layers. The Ca/P ratio in the superficial layer was significantly lower compared with the other two layers.

### Study 3: Elemental analysis comparing small and large permanent tusks

A total of 20 elements plus LE were detected in small and large dugong tusks (Table 3). Three elements were significantly higher in small tusks, including P, Ca and Zr, while Fe, Pb, LE and Ca/P ratio was significantly higher in large tusks.

### Study 4: Elemental analysis comparing dugong sexes

A total of 20 elements plus LE were detected in tusks from both sexes (Table 4). Only one element (Ag) showed a significant difference between sexes (higher in males than females). LE in females were significantly higher than in males. The elemental composition of dugong tusks was used for creating an equation for assigning sex, as follows:


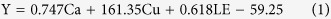


**Figure 2:**
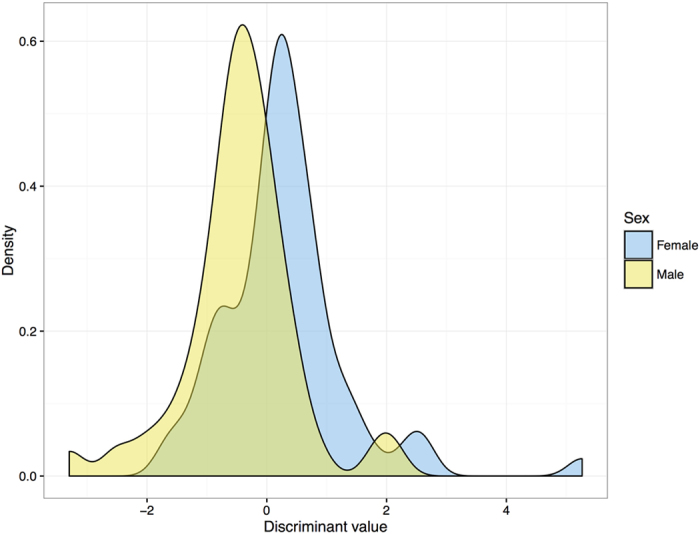
The distribution of discriminant values between male and female dugong tusks based on the elemental composition.

As shown in Fig. 2, in the distribution of discriminant values by gender obtained from the equation, there was a large overlap between males and females. Correct prediction of male dugong tusks was relatively low, 68.8%, about the same as for females (68.6%). The overall accuracy rate, 68.9%, was moderate (Table 5).

### Study 5: Elemental analysis comparing dugong tusks from the Andaman Sea and the Gulf of Thailand

A total of 20 elements plus LE were detected in tusks taken from different habitats (Table 6). There were two elements (Al and W) which were not found in dugong tusks from the Gulf of Thailand. Sulfur (S) was significantly higher in dugong tusks from the Andaman Sea, while titanium (Ti) was significantly higher in dugong tusks from the Gulf of Thailand. The equation for estimating dugong location based on the elemental composition of tusks is given by:


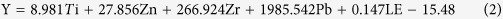


**Figure 3:**
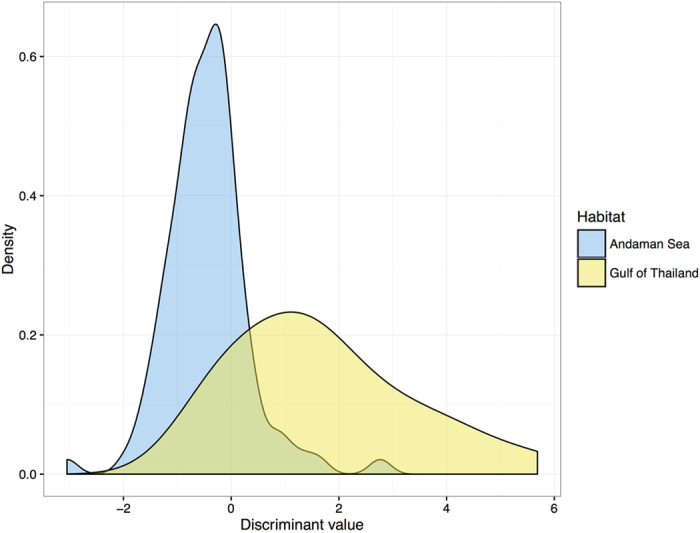
The distribution of discriminant values between the Andaman Sea and the Gulf of Thailand based on the elemental composition of dugong tusks.

An overlapping area of discriminant values obtained from the Andaman Sea and the Gulf of Thailand was observed (Fig. 3). We found that this equation could correctly identify dugongs from the Andaman Sea with high accuracy, 93.7%, but with a low level of correct prediction for dugongs from the Gulf of Thailand (Table 7). The overall accuracy for estimating location was 88.1%.

### Study 6: Elemental analysis comparing dugong tusks, and dugong, dolphin and whale teeth

**Figure 4:**
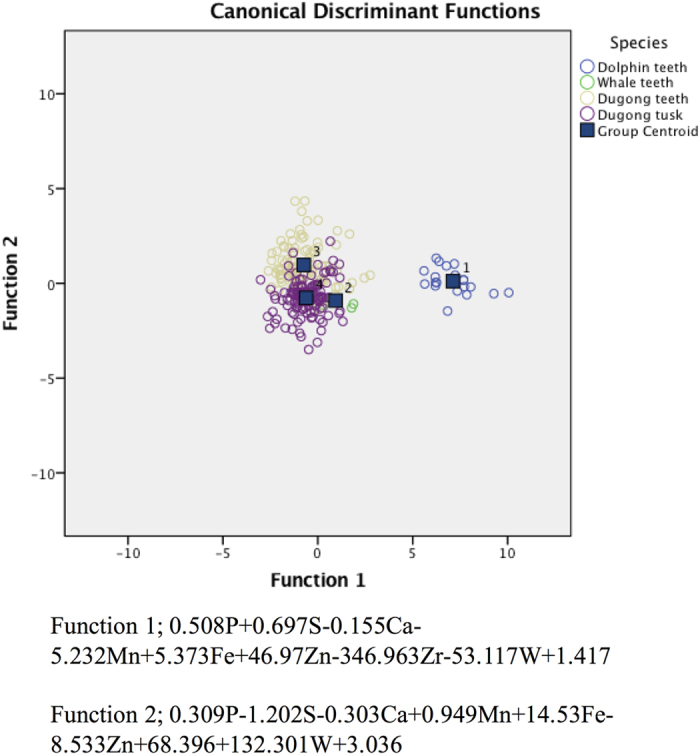
The feasibility of species classification by the elemental composition of tusks and teeth. Canconical discriminant function plots of the elemental composition of teeth and tusks of different species. P = phosphorus, S = sulfur, Ca = calcium, Mn = manganese, Fe = iron, Zn = zinc, Zr = zirconium, W = tungsten.

A total of 20 elements plus LE were detected in dugong tusks, and dugong, dolphin and whale teeth (Table 8). We found that for two elements, nickel (Ni) and lead (Pb), there was not a significant difference between the four tooth types. The Ca/P ratio was significantly higher in dugong tusks, and significantly lowest in dolphin teeth. We created an equation for differentiating dugong tusks and dugong, dolphin and whale teeth based on their elemental composition, as shown in Fig. 4. Dolphin teeth were able to be correctly predicted 100% of the time, followed by dugong tusks, dugong and whale teeth, with correct prediction rates of 79.3%, 75.3% and 75%, respectively (Table 9). The whale tooth was misidentified as a dugong tusk 25% of the time (Table 9). The overall accuracy of differentiating species by this equation was 79.3% (Table 9).

### Study 7: Elemental analysis comparing dugong tusks, Asian elephant tusks and tiger canine teeth

To differentiate among dugong and Asian elephant tusks and tiger canine teeth, their elemental composition was used to establish an effective equation using stepwise discriminant analysis.

**Figure 5:**
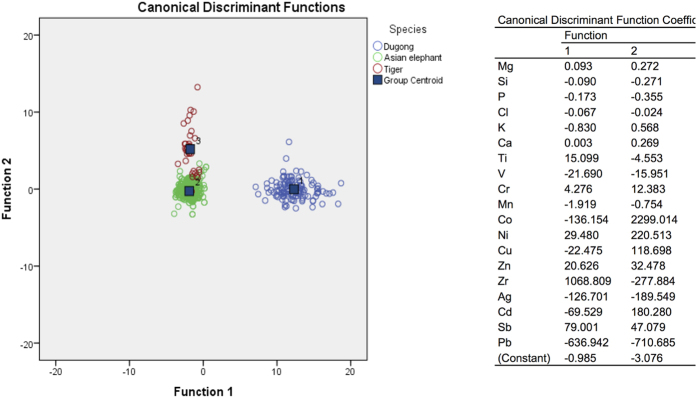
The feasibility of distinguishing three species (dugong, Asian elephant and tiger) by the elemental composition of tusks and teeth. Canonical discriminant function plots of the elemental composition of dugong tusks, Asian elephant tusks and tiger canine teeth. Mg = magnesium, Si = silicon, P = phosphorus, Cl = chlorine, K = potassium, Ca = calcium, Ti = titanium, V = vanadium, Cr = chromium, Mn = manganese, Co = cobalt, Ni = nickel, Cu = copper, Zn = zinc, Zr = zirconium, Ag = silver, Sb = antimony.

As shown in Fig. 5, a discrepancy of discriminant values between dugong tusks and teeth, Asian elephant tusks and tiger canine teeth was noted, indicating a high correct prediction rate among species of 98.2% (Table 10). However, there was a small misclassification within tiger canines, which were predicted as Asian elephant and dugong tusks 26.3% and 2.6% of the time, respectively.

## Discussion

When we compared the elements found in dugong teeth and tusks using a HH-XRF with either tusks or teeth of other species, the data obtained consisted of four important points. (i) Three major elements with quantity > 1%, i.e. Ca, P and Si, could be detected in dugong tusks and teeth, while Al was found only in dugongs (at a low level) but in no other species. (ii) Elemental analysis can likely be used for differentiating dugong tusks from different habitats, with a high accuracy rate (88.1%). (iii) It was feasible to distinguish dugong teeth from two other marine mammal species (dolphin and whale), with a 79.3% accuracy rate. (iv) Moreover, dugong tusks could be distinguished from elephant tusks and tiger canine teeth with 100% accuracy by elemental analysis.

### Elements in dugong tusks

From studies 1–3, it was shown that the distribution of elements was not even in tusk samples. Most of the elements accumulated in a greater amount at the surface of the crown as compared with the root. However, there was no difference in the distribution of the major elements (Ca, P, and Ca/P ratio). A comparison of the three different layers of dugong tusks revealed that most elements showed the highest accumulation in the surface layer, whereas the Ca/P ratio was lowest in the superficial layer. These results were in contrast with a study of the elements in human, bovine, porcine and ovine teeth, in which the Ca/P ratio was higher in the superficial (enamel) layer than in the dentine layer^26^. However, the surface area of dugong tusks is not covered with enamel, as in other species; the enamel covers only the tips of tusks when dugongs are young, but the enamel disappears in older animals^4^. It is possible that because of the continued growth of tusks from inside to outside throughout life^4^, the change of elemental accumulation in the superficial layer occurs over a longer period than in the medial layer.

The Ca/P ratio is representative of calcium hydroxyapatite and is indicative of the strength of bones and teeth^16^,^17^,^38^,^39^. There was no difference in the Ca/P ratio between the crown and root of dugong tusks, but it was significantly higher in the intermediate and medial layers compared with the superficial layer. However, we found that large tusks had a significantly higher Ca/P ratio than small tusks. Moreover, dugong tusks had a higher Ca/P ratio than the other three marine tissues studied (dugong teeth, dolphin teeth and whale tooth).

### Elements and the estimation of sex and habitat

In this study, we analyzed the elemental content in tusks for two main purposes: (i) sex and (ii) habitat estimation. We found that most of the elements were not significantly different between sexes, except Ag which was significantly higher in males. Silver is one of the heavy metals and can be toxic to aquatic animals^43^. However, we cannot explain why Ag was higher in male dugong tusks. According to some reports in the literature, it is believed that gender slightly influences the accumulation of heavy metals in the body^18^,^31^,^44^. However, it was previously demonstrated in striped dolphin (*Stenella coeruleoalba*) that gender exhibited little effect on the accumulation of some heavy metals (Fe, Mn, Zn, Cu, Pb, Ni, Cd and Hg) in body tissues^44^. Moreover, in a recent study of 47 canine compact bone types, little relation was found between gender and the accumulation of heavy metals^31^. The best equation for determining sex based on the data of elemental content in dugong tusks provided a moderate accuracy rate of 68.9%. This result indicated that elemental content may not be pertinent for assigning sex in dugongs. Also, the elemental content in male and female elephant tusks is not much different^25^. In humans, the elemental content in bone (cranium, humerus, os coxae and their combination) has been used for sex classification, but it only gave an accuracy rate ranging from 60 to 67%^21^. Taken together, we have concluded that elemental analysis might not be a worthy tool for sex identification in dugongs.

In addition, we used the elemental content of dugong tusks for the estimation of the location of the dugongs’ habitat. It was found that Al and W were present in dugong tusks from the Andaman Sea, and that there was a significant difference in S and Ti content between tusks from the Andaman Sea and the Gulf of Thailand. The differences in these four elements (Al, W, S and Ti) might be due to industrial contamination of the coastline, which is the habitat of dugongs, or because some elements are found in a particular area. For instance, W is a rare earth mineral which is found in some areas of Thailand^45^. It is possible that these elements accumulated in seagrass, which is the main food of dugongs^46^,^47^,^48^. An equation for discriminating dugong tusks from the Andaman Sea and the Gulf of Thailand showed an accuracy rate of 88.1%. Previously, our team utilized elemental content to estimate the origin of elephant tusks (Asia or Africa) with an accuracy rate of 94%^25^. This evidence demonstrates that elemental content could serve as an effective method for differentiating the origin or habitat of animals.

### Elements and species identification

Aluminum was detected in dugongs only (both teeth and tusks), but not in dolphin and whale teeth. In a recent study of the elemental distribution in eight species (deer, dog, elephant, horse, human, monkey, dolphin and crocodile), Al was detected in elephant, human and monkey teeth^24^. This suggests that Al might be involved in some physiological pathway of dugongs, elephants, humans and monkeys. Dugongs and elephants belong to the same clade, Tethytheria, while humans and monkeys belong to the same order, Primatomorpha. To clarify our hypothesis, further work should be done.

In comparing dugong tusks, and dugong, dolphin and whale teeth, there was a correct prediction rate of 100% for dolphin teeth. Dugong tusks were correctly predicted 79.3% of the time; the misclassification rate was 20.7%, consisting of 15.7% predicted as dugong teeth and 5% predicted as whale tooth. A previous study showed an accuracy rate of 78.4% for identifying eight tooth species based on their elemental content^24^. Furthermore, the elemental content among dugong tusks, Asian elephant tusks and tiger canine teeth could be differentiated with an overall accuracy rate of 98.2%. We found that dugong tusks could be completely discriminated from Asian elephant tusks and tiger canine teeth, while some Asian elephant tusks were predicted as tiger teeth (0.7%) and some tiger teeth were predicted as dugong tusks (2.6%) and Asian elephant tusks (26.3%). It is plausible that dugongs and the other two animals have a significantly distinct habitat (land and marine), resulting in a clear difference in elemental composition in spite of the fact that they were scanned at the same region (upper incisor) in both species. Similarly, a previous study demonstrated that the identification of tusks from elephants living on different continents, Asia and Africa, also showed a high accuracy rate of 94%^25^.

### Limitations

There were some limitations of this study. HH-XRF equipment is convenient to transport and use in the field; however, it only scans elements at the surface of a sample. Thus, if the surface is treated with chemical agents or protectants that contain elements detected by XRF, the percentages could be altered and lead to inaccurate measurements and false identification. For this reason, all samples were untreated and cleaned of any dirt and debris before scanning. The HH-XRF machine does not provide probing volumes, so we could not calculate elemental concentrations; rather, these were presented as percentages. Elemental composition was compared through stepwise discriminant analysis. Light elements in samples that were not quantifiable by HH-XRF analysis had an impact on some elemental proportions, so the use of methodology to quantify these components might provide additional information that could lead to further improvements in discrimination functions. Finally, the number of samples from some species, whales in particular, was low because of the rarity of samples.

## Conclusion

The elemental composition of biological materials is useful in a wide range of sciences. Our results have demonstrated the utilization of elements in tusks and teeth for differentiating animal species. In addition, we observed that elemental analysis of tusks and teeth could not be used to distinguish between male and female dugongs. In contrast, elemental analysis was shown to be an excellent candidate tool for estimation of animal habitat, as it could distinguish dugong tusks from the Andaman Sea and the Gulf of Thailand. These results may be beneficial for law enforcement authorities in resolving the difficulty of identification of tusks. Finally, we described the potential feasibility of utilizing handheld XRF to examine tusks and teeth for the preliminary determination of species, prior to using more advanced techniques such as molecular biology.

## Additional Information

**How to cite this article**: Nganvongpanit, K. *et al*. Elemental classification of the tusks of dugong (Dugong dugong) by HH-XRF analysis and comparison with other species. *Sci. Rep.* **7**, 46167; doi: 10.1038/srep46167 (2017).

**Publisher's note:** Springer Nature remains neutral with regard to jurisdictional claims in published maps and institutional affiliations.

## Figures and Tables

**Table 1:** Percentage of elements detected and Ca/P ratio in the crown and root of dugong tusks.

Ele.	Crown	Root	p-value	Ele.	Crown	Root	p-value
Al	0.324 ± 0.240	0.237 ± 0.0017	0.767	Cu	0.032 ± 0.092	0.016 ± 0.037	0.598
Si	1.252 ± 1.420	1.247 ± 1.305	0.753	Zn	0.042 ± 0.014	0.038 ± 0.013	0.090
P	8.016 ± 1.656	8.206 ± 1.885	0.710	Zr	0.013 ± 0.003	0.014 ± 0.002	0.501
S	0.356 ± 0.417	0.366 ± 0.491	0.773	Ag	**0.019** ± **0.003**	0.017 ± 0.004	0.043
Ca	23.219 ± 3.509	24.122 ± 3.512	0.404	Cd	0.024 ± 0.005	0.022 ± 0.006	0.053
Ti	**0.066** ± **0.053**	0.048 ± 0.030	0.000	Sn	**0.027** ± **0.006**	0.024 ± 0.007	0.011
V	**0.019** ± **0.004**	0.018 ± 0.004	0.023	Sb	**0.036** ± **0.008**	0.032 ± 0.009	0.013
Cr	0.015 ± 0.026	0.010 ± 0.003	0.282	W	0.004 ± 0.001	0.004 ± 0.001	0.694
Mn	**0.015** ± **0.030**	0.009 ± 0.003	0.001	Pb	0.001 ± 0.001	0.001 ± 0.001	0.921
Fe	**0.050** ± **0.036**	0.030 ± 0017	0.000	LE	66.783 ± 5.667	65.803 ± 5.625	0.532
Ni	0.002 ± 0.00	0.002 ± 0.001	0.864	Ca/P	2.896 ± 0.375	3.027 ± 0.440	0.519

Bold numbers indicate significantly higher values. Al = aluminum, Si = silicon, P = phosphorus, S = sulfur, Ca = calcium, Ti = titanium, V = vanadium, Cr = chromium, Mn = manganese, Fe = iron, Ni = nickel, Cu = copper, Zn = zinc, Zr = zirconium, Ag = silver, Cd = cadmium, Sn = tin, Sb = antimony, W = tungsten, Pb = lead, LE = light elements.

**Table 2:** Percentage of elements detected and Ca/P ratio in superficial, intermediate and medial layers of dugong tusks.

Ele.	Superficial	Intermediate	Medial	p-value
Al	0.203 ± 0.061^a^	1.337 ± 0.275^b^	1.873 ± 0.215^c^	0.027
Si	**2.125** ± **1.457**^**a**^	0.416 ± 0.213^b^	0.347 ± 0.220^b^	0.000
P	**7.393** ± **1.137**^**a**^	3.252 ± 1.637^b^	3.601 ± 1.691^b^	0.000
S	0.203 ± 0.113^a^	**0.282** ± **0.076**^**b**^	0.275 ± 0.086^b^	0.014
Cl	N.D.	2.741 ± 1.184	2.597 ± ± 0.532	0.124
K	N.D.	0.194 ± 0.126	0.123 ± 0.052	0.850
Ca	**22.137 ± 2.142**^**a**^	14.215 ± 3.466^b^	15.324 ± 2.594^b^	0.000
Ti	**0.045 ± 0.015**^**a**^	0.032 ± 0.007^b^	0.031 ± 0.006^b^	0.000
V	**0.018 ± 0.003**^**a**^	0.015 ± 0.003^b^	0.016 ± 0.004^a,b^	0.029
Cr	**0.009 ± 0.002**^**a**^	0.008 ± 0.002^a,b^	0.008 ± 0.001^b^	0.024
Mn	**0.008 ± 0.002**^**a**^	0.006 ± 0.001^b^	0.007 ± 0.001^b^	0.000
Fe	**0.039 ± 0.031**^**a**^	0.016 ± 0.003^b^	0.017 ± 0.005^b^	0.000
Ni	0.002 ± 0.000	0.003 ± 0.007	0.002 ± 0.000	0.338
Cu	0.005 ± 0.007	0.003 ± 0.002	0.002 ± 0.000	0.778
Zn	**0.037 ± 0.009^a^**	0.013 ± 0.003^b^	0.014 ± 0.004^c^	0.000
Zr	0.013 ± 0.002	0.013 ± 0.002	0.014 ± 0.004	0.601
Ag	**0.016 ± 0.002**^**a**^	0.014 ± 0.002^b^	0.015 ± 0.001^b^	0.004
Cd	**0.021 ± 0.003**^**a**^	0.019 ± 0.002^b^	0.020 ± 0.002^b^	0.001
Sn	**0.024 ± 0.004**^**a**^	0.023 ± 0.002^b^	0.022 ± 0.002^b^	0.008
Sb	**0.031 ± 0.005**^**a**^	0.030 ± 0.002^a,b^	0.030 ± 0.003^b^	0.037
W	0.003 ± 0.000	N.D.	N.D.	N.A.
Pb	0.001 ± 0.001	N.D.	N.D.	N.A.
LE	67.857 ± 3.436^a^	**80.452 ± 3.726**^**b**^	79.217 ± 3.724^b^	0.000
Ca/P	3.051 ± 0.359^a^	**5.049 ± 1.931**^**b**^	**5.049 ± 1.931**^**b**^	0.000

Bold numbers indicate significantly higher values. N.D. = not detected, N.A. = not analyzed. Al = aluminum, Si = silicon, P = phosphorus, S = sulfur, Cl = chlorine, K = potassium, Ca = calcium, Ti = titanium, V = vanadium, Cr = chromium, Mn = manganese, Fe = iron, Ni = nickel, Cu = copper, Zn = zinc, Zr = zirconium, Ag = silver, Cd = cadmium, Sn = tin, Sb = antimony, W = tungsten, Pb = lead, LE = light elements. ^a,b,c^Indicate a significant difference at p < 0.05 when compared in the different layers of dugong tusks

**Table 3:** Percentage of elements detected and Ca/P ratio in small and large dugong tusks.

Ele.	Small	Large	p-value	Ele.	Small	Large	p-value
Al	0.361 ± 0.327	0.203 ± 0.061	1.000	Cu	0.046 ± 0.100	0.004 ± 0.006	0.059
Si	1.611 ± 1.854	0.901 ± 0.941	0.914	Zn	0.043 ± 0.015	0.039 ± 0.012	0.098
P	**9.190 ± 1.907**	7.642 ± 1.388	0.000	Zr	**0.015 ± 0.002**	0.013 ± 0.002	0.001
S	0.326 ± 0.419	0.262 ± 0.308	0.882	Ag	0.018 ± 0.004	0.018 ± 0.003	0.811
Ca	**25.804 ± 3.688**	22.571 ± 2.881	0.000	Cd	0.022 ± 0.007	0.023 ± 0.005	0.526
Ti	0.054 ± 0.054	0.058 ± 0.036	0.063	Sn	0.024 ± 0.008	0.026 ± 0.006	0.307
V	0.018 ± 0.004	0.018 ± 0.004	0.305	Sb	0.031 ± 0.011	0.035 ± 0.007	0.226
Cr	0.010 ± 0.002	0.014 ± 0.022	0.586	W	0.004 ± 0.001	0.004 ± 0.000	0.858
Mn	0.009 ± 0.003	0.013 ± 0.027	0.432	Pb	0.001 ± 0.000	**0.001 ± 0.001**	0.028
Fe	0.028 ± 0.013	**0.045 ± 0.034**	0.000	LE	62.785 ± 6.265	**68.293 ± 4.209**	0.000
Ni	0.002 ± 0.000	0.002 ± 0.001	0.141	Ca/P	2.867 ± 0.341	**3.007 ± 0.435**	0.015

Bold numbers indicate significantly higher values. Al = aluminum, Si = silicon, P = phosphorus, S = sulfur, Ca = calcium, Ti = titanium, V = vanadium, Cr = chromium, Mn = manganese, Fe = iron, Ni = nickel, Cu = copper, Zn = zinc, Zr = zirconium, Ag = silver, Cd = cadmium, Sn = tin, Sb = antimony, W = tungsten, Pb = lead, LE = light elements.

**Table 4:** Percentage of elements detected and Ca/P ratio in male and female dugong tusks.

Ele	Male	Female	p-value	Ele.	Male	Female	p-value
Al	0.125 ± 0.007	3.00 ± 0.201	0.157	Cu	0.002 ± 0.000	0.008 ± 0.012	0.696
Si	1.2226 ± 1.289	1.033 ± 1.229	0.257	Zn	0.040 ± 0.015	0.041 ± 0.012	0.544
P	8.331 ± 1.753	7.849 ± 1.668	0.070	Zr	0.013 ± 0.002	0.014 ± 0.002	0.733
S	0.355 ± 0.434	0.289 ± 0.408	0.964	Ag	**0.019 ± 0.003**	0.017 ± 0.004	0.029
Ca	23.990 ± 3.690	23.161 ± 3.230	0.064	Cd	0.023 ± 0.006	0.022 ± 0.005	0.389
Ti	0.057 ± 0.052	0.057 ± 0.037	0.852	Sn	0.026 ± 0.007	0.026 ± 0.006	0.337
V	0.018 ± 0.004	0.018 ± 0.004	0.820	Sb	0.035 ± 0.009	0.033 ± 0.009	0.173
Cr	0.016 ± 0.027	0.010 ± 0.002	0.092	W	0.004 ± 0.001	0.004 ± 0.001	1.000
Mn	0.015 ± 0.032	0.009 ± 0.002	0.265	Pb	0.001 ± 0.000	0.001 ± 0.000	0.166
Fe	0.038 ± 0.031	0.039 ± 0.028	0.282	LE	65.778 ± 5.543	**67.376 ± 5.198**	0.030
Ni	0.02 ± 0.001	0.002 ± 0.001	0.801	Ca/P	2.939 ± 0.405	3.017 ± 0.432	0.259

Bold numbers indicate significantly higher values. Al = aluminum, Si = silicon, P = phosphorus, S = sulfur, Ca = calcium, Ti = titanium, V = vanadium, Cr = chromium, Mn = manganese, Fe = iron, Ni = nickel, Cu = copper, Zn = zinc, Zr = zirconium, Ag = silver, Cd = cadmium, Sn = tin, Sb = antimony, W = tungsten, Pb = lead, LE = light elements.

**Table 5:** Classification result of identifying gender using the equation obtained from elemental composition.

Sex	Prediction		Accuracy^a^
	Male	Female	
Male	68.8	31.3	68.9
Female	31.4	68.6	

^a^Cross-validation is done only for those cases in the analysis. In cross-validation, each case is classified by the functions derived from all cases other than that case.

**Table 6:** Percentage of elements detected and Ca/P ratio in dugong tusks from the Andaman Sea and the Gulf of Thailand.

Ele.	Andaman Sea	Gulf of Thailand	p-value	Ele.	Andaman Sea	Gulf of Thailand	p-value
Al	0.300 ± 0.201	N.D.	N.D.	Cu	0.007 ± 0.011	0.003 ± 0.003	0.234
Si	1.183 ± 1.334	0.669 ± 0.641	0.080	Zn	0.039 ± 0.013	0.046 ± 0.013	0.062
P	8.056 ± 1.680	7.713 ± 1.680	0.320	Zr	0.013 ± 0.002	0.014 ± 0.002	0.469
S	**0.339 ± 0.429**	0.116 ± 0.064	0.026	Ag	0.018 ± 0.003	0.018 ± 0.004	0.905
Ca	23.383 ± 3.282	23.134 ± 3.282	0.783	Cd	0.023 ± 0.006	0.023 ± 0.004	0.997
Ti	0.049 ± 0.022	**0.092 ± 0.084**	0.007	Sn	0.026 ± 0.007	0.027 ± 0.006	0.908
V	0.018 ± 0.004	0.018 ± 0.003	0.608	Sb	0.034 ± 0.009	0.035 ± 0.007	0.757
Cr	0.013 ± 0.020	0.010 ± 0.002	0.784	W	0.004 ± 0.001	N.D.	N.A.
Mn	0.012 ± 0.024	0.010 ± 0.003	0.939	Pb	0.001 ± 0.000	0.001 ± 0.000	0.263
Fe	0.040 ± 0.032	0.033 ± 0.018	0.523	LE	66.734 ± 5.105	68.047 ± 5.104	0.279
Ni	0.002 ± 0.001	0.003 ± 0.001	0.227	Ca/P	2.968 ± 0.435	3.064 ± 0.383	0.149

Bold numbers indicate significantly higher values. N.D. = not detected, N.A. = not analyzed. Al = aluminum, Si = silicon, P = phosphorus, S = sulfur, Ca = calcium, Ti = titanium, V = vanadium, Cr = chromium, Mn = manganese, Fe = iron, Ni = nickel, Cu = copper, Zn = zinc, Zr = zirconium, Ag = silver, Cd = cadmium, Sn = tin, Sb = antimony, W = tungsten, Pb = lead, LE = light elements.

**Table 7:** Classification result of estimating location using the equation obtained from elemental composition.

Location	Prediction		Accuracy^a^
	Andaman Sea	Gulf of Thailand	
Andaman Sea	93.7	6.3	88.1
Gulf of Thailand	34.8	65.2	

^a^Cross-validation is done only for those cases in the analysis. In cross-validation, each case is classified by the functions derived from all cases other than that case.

**Table 8:** Percentage of elements detected and Ca/P ratio in dugong tusks, and dugong, dolphin and whale teeth.

Ele.	Dugong tusks	Dugong teeth	Dolphin teeth	Whale tooth	p-value
Al	0.266 ± 0.190	0.317 ± 0.084	N.D.	N.D.	N.D.
Si	**1.114 ± 1.253**^**a**^	0.373 ± 0.212^a^	0.178 ± 0.037^b^	0.465 ± 0.174^a^	0.000
P	8.052 ± 1.714^a^	7.559 ± 1.219^b^	**9.286 ± 0.617**^**c**^	8.119 ± 0.811^a,b^	0.000
S	**0.316 ± 0.418**^**a**^	0.086 ± 0.039^b^	0.049 ± 0.032^c^	0.271 ± 0.071^d^	0.000
Ca	**23.510 ± 3.441**^**a**^	21.217 ± 1.910^b^	20.233 ± 1.540^c^	22.798 ± 0.924^d^	0.004
Ti	**0.057 ± 0.044**^**a**^	0.047 ± 0.020^b^	0.052 ± 0.011^c^	0.036 ± 0.010^a,b^	0.020
V	**0.018 ± 0.004**^**a**^	0.017 ± 0.005^a^	0.013 ± 0.003^b^	0.014 ± 0.002^a,b^	0.010
Cr	**0.012 ± 0.018**^**a**^	0.009 ± 0.002^b^	0.007 ± 0.002^c^	0.010 ± 0.001^d^	0.000
Mn	0.012 ± 0.021^a^	**0.058 ± 0.089**^**b**^	0.015 ± 0.008^c^	0.008 ± 0.001^a^	0.000
Fe	0.039 ± 0.030^a^	**0.097 ± 0.065**^**b**^	0.072 ± 0.032^b^	0.019 ± 0.002^c^	0.003
Ni	0.002 ± 0.001	0.002 ± 0.001	0.002 ± 0.000	0.002 ± 0.000	0.789
Cu	**0.006 ± 0.010**^**a**^	0.002 ± 0.000^b^	0.002 ± 0.000^b^	0.002 ± 0.000^b^	0.002
Zn	0.040 ± 0.013^a^	0.038 ± 0.017^b^	**0.094 ± 0.029**^**c**^	0.035 ± 0.009^b,d^	0.000
Zr	0.014 ± 0.002^a^	**0.014 ± 0.003**^**b**^	0.002 ± 0.000^c^	0.008 ± 0.002 ^a,c^	0.000
Ag	**0.018 ± 0.003**^**a**^	0.017 ± 0.004^a^	0.014 ± 0.002^b^	0.015 ± 0.001^a,b^	0.003
Cd	**0.023 ± 0.006**^**a**^	**0.023 ± 0.005**^**a**^	0.017 ± 0.004^b^	0.021 ± 0.001^a^	0.000
Sn	0.026 ± 0.007^a^	**0.027 ± 0.006**^**a**^	0.019 ± 0.005^b^	0.023 ± 0.001^a,b^	0.000
Sb	**0.034 ± 0.009**^**a**^	**0.034 ± 0.007**^**a**^	0.025 ± 0.007^b^	0.032 ± 0.001^a,c^	0.000
W	0.004 ± 0.001	0.003 ± 0.001	N.D.	0.004 ± 0.000	0.064
Pb	0.001 ± 0.001	0.001 ± 0.001	0.001 ± 0.000	0.001 ± 0.000	0.365
LE	66.702 ± 5.382^a^	**70.013 ± 2.823**^**b**^	69.942 ± 2.098^b^	68.123 ± 1.603^a,b^	0.000
Ca/P	**2.984 ± 0.421**^**a**^	2.862 ± 0.422^b^	2.179 ± 0.075^c^	2.825 ± 0.247^a,b,d^	0.000

Bold numbers indicate significantly higher values. N.D. = not detected. Al = aluminum, Si = silicon, P = phosphorus, S = sulfur, Ca = calcium, Ti = titanium, V = vanadium, Cr = chromium, Mn = manganese, Fe = iron, Ni = nickel, Cu = copper, Zn = zinc, Zr = zirconium, Ag = silver, Cd = cadmium, Sn = tin, Sb = antimony, W = tungsten, Pb = lead, LE = light elements. ^a,b,c,d^ indicate a significant difference at p < 0.05 when compared across dugong tusks, and dugong, dolphin and whale teeth.

**Table 9:** Classification result of species prediction using the equation obtained from elemental composition.

Species	Prediction				Accuracy^a^
	Dolphin teeth	Whale tooth	Dugong teeth	Dugong tusks	
Dolphin teeth	100	0	0	0	79.3
Whale tooth	0	75	0	25	
Dugong teeth	0	9.3	75.3	15.4	
Dugong tusks	0	5	15.7	79.3	

^a^Cross-validation is done only for those cases in the analysis. In cross-validation, each case is classified by the functions derived from all cases other than that case.

**Table 10:** Classification result of tusk species prediction using the equation obtained from elemental composition.

Species	Prediction			Accuracy^a^
	Dugong tusks	Asian elephant tusks	Tiger teeth	
Dugong tusks	100.0	0.0	0.0	98.2
Asian elephant tusks	0.0	99.3	0.7	
Tiger teeth	2.6	26.3	71.1	

^a^Cross-validation is done only for those cases in the analysis. In cross-validation, each case is classified by the functions derived from all cases other than that case.
